# The Clinical Significance of DJ1 and L1CAM Serum Level Monitoring in Patients with Endometrial Cancer

**DOI:** 10.3390/jcm10122640

**Published:** 2021-06-15

**Authors:** Marketa Bednarikova, Petra Vinklerova, Jana Gottwaldova, Petra Ovesna, Jitka Hausnerova, Lubos Minar, Michal Felsinger, Dalibor Valik, Zdenka Cermakova, Vit Weinberger

**Affiliations:** 1 Department of Internal Medicine, Hematology and Oncology, Masaryk University and University Hospital, 625 00 Brno, Czech Republic; bednarikova.marketa@fnbrno.cz; 2 Department of Obstetrics and Gynecology, Masaryk University and University Hospital, 625 00 Brno, Czech Republic; vinklerova.petra@fnbrno.cz (P.V.); minar.lubos@fnbrno.cz (L.M.); felsinger.michal@fnbrno.cz (M.F.); 3 Department of Laboratory Medicine, Department of Laboratory Methods, Masaryk University and University Hospital, 625 00 Brno, Czech Republic; jana.gottwaldova@mou.cz (J.G.); valik.dalibor@fnbrno.cz (D.V.); zdenka.cermakova@mou.cz (Z.C.); 4 Department of Laboratory Medicine, Masaryk Memorial Cancer Institute, 602 00 Brno, Czech Republic; 5 Faculty of Medicine, Institute of Biostatistics and Analyses, Masaryk University, 625 00 Brno, Czech Republic; ovesna@iba.muni.cz; 6 Department of Pathology, Masaryk University and University Hospital, 625 00 Brno, Czech Republic; hausnerova.jitka@fnbrno.cz

**Keywords:** tumor markers, endometrial cancer, DJ1, L1CAM

## Abstract

Circulating tumor markers are not routinely used in patients with endometrial cancer (EC). This pilot study evaluated the role of monitoring new biomarkers DJ1 and L1CAM, in correlation with CA125 and HE4, for the effects of anticancer treatment and preoperative management in EC patients. Serial serum levels of DJ1, L1CAM, CA125 and HE4 were collected in 65 enrolled patients. Serum DJ1, L1CAM, CA125 and HE4 levels were significantly higher at the time of diagnosis compared to those measured during follow-up (FU). In patients with recurrent disease, serum DJ1, CA125 and HE4 levels were significantly higher at the time of recurrence compared to levels in disease-free patients. Serum L1CAM levels were also higher in patients with recurrence but without reaching statistical significance. While DJ1 levels were not affected by any of the observed patient-related characteristics, L1CAM levels were significantly higher in patients with age ≥60 years who were overweight. At the time of EC diagnosis, DJ1 and L1CAM serum levels did not correlate with stage, histological type or risk of recurrence. This is a preliminary description of the potential of serial DJ1 and L1CAM serum level measurement for monitoring the effects of treatment in EC patients.

## 1. Introduction

The primary clinical use of circulating tumor markers determined in peripheral blood consists of monitoring the course of disease. An increase in serum levels often precedes the clinical manifestation of disease recurrence, and dynamic changes in their levels over time are used to monitor the effects of anticancer treatments. In contrast, circulating markers are not very suitable for cancer screening or primary diagnosis and do not play a significant role in determining the extent of the disease or prognosis, respectively [1,2].

In endometrial cancer (EC) patients, elevated CA125 and HE4 levels have been frequently found [3]. The results of studies investigating the prognostic impact of CA125 in EC have not been unequivocal. While Sood et al. and Reijnen et al. described an association between preoperatively elevated CA125 levels and poor outcomes in EC patients [4,5], other studies did not confirm the correlation between CA125 serum levels and extent of disease at the time of diagnosis [6,7].

In terms of EC detection, marker HE4 has demonstrated higher sensitivity and specificity than CA125, especially in early stages [8]. The prognostic relevance of HE4 has been established in poorly differentiated EC [9]. Neither of the circulating markers have become a standard part of clinical practice [10,11,12,13].

Recently, studies of new promising markers DJ1 and L1CAM have been published. The first marker, DJ1, also known as Parkinson’s disease-associated protein 7 (PARK7), is a multifunctional protein promoting cell proliferation and playing an important role in cancer pathogenesis and progression by modulating the tumor suppressor PTEN. The results of studies by Italian authors demonstrated not only significantly higher DJ1 serum levels in EC patients compared to healthy controls, but also the association of higher DJ1 levels with high-risk histological type (defined as endometrioid carcinoma grade 3 or non-endometrioid types) in contrast with lower DJ1 levels in low-risk histological types (endometrioid carcinoma grade 1 or 2) [14,15,16]. The second marker, L1 cell adhesion molecule (L1CAM), is a membrane glycoprotein of the immunoglobulin family, crucially involved in cancer cell migration and adhesion. L1CAM overexpression in EC tissues represents a negative prognostic marker, signaling both more aggressive behavior of the tumor and shorter survival of patients [17,18]. The significance of L1CAM serum level measurement has not been unambiguously established yet [19,20]. All studies concerning circulating DJ1 and L1CAM serum levels assessed merely one-time sampling events without studying the potential significance of time-dependent changes in serial DJ1 or L1CAM serum levels. Therefore, data regarding the importance of DJ1 and L1CAM measurement either during the course of therapy for the monitoring of their effects or during a follow-up after successful primary therapy for the detection of EC recurrence are still lacking.

The aim of our study was to evaluate whether time-dependent changes of serial serum measurements of DJ1, L1CAM, CA125 and HE4 in EC patients correlated with the course of the disease and whether elevated levels at the follow-up signalized recurrence. We wanted to clarify whether the markers’ levels were affected by factors associated with patients’ health conditions. We also investigated if marker levels at the time of diagnosis correlated with clinico-pathological features of the tumor.

## 2. Materials and Methods

### 2.1. Patients

Patients undergoing surgical treatment from May 2016 to April 2019 for histologically proven EC in the Oncogynecological Center of University Hospital (UH) Brno, Czech Republic, were consecutively involved in this single-institutional, prospective, observational study. The patients with other malignancies or neuro-degenerative diseases were excluded. All subjects gave their written informed consent to participate in the study.

### 2.2. Clinical Management

Diagnosis of EC was made after histopathological examination of a tumor biopsy obtained from hysteroscopy or dilatation and curettage. Each patient with newly diagnosed EC underwent a clinical examination, CT of the chest/abdomen and an expert ultrasound (US) examination, according to local guidelines [21]. The blood samples for CA125, HE4, DJ1 and L1CAM serum level assessment as well as for basic biochemical and hematological laboratory tests were performed at the time of diagnosis. Subsequently, patients were divided into groups with a low or high risk of recurrence according to histology, grade and clinical staging. The low-risk group was defined as endometrioid carcinoma grade 1 TNM stage cT1a or cT1b and/or endometrioid carcinoma grade 2 TNM stage cT1a, all of them without clinical or imaging evidence of lymphadenopathy (cN0) or distant metastases (cM0) [22]. Patients who did not meet criteria for a low-risk group were classified as a high-risk group. The staging surgical procedure consisting of a total hysterectomy with bilateral salpingo-oophorectomy was performed in all patients. Whereas the sentinel node biopsy was not a standard at the time of the study in our department, systematic pelvic and paraaortic lymphadenectomies were performed in the high-risk group. In the case of serous endometrial carcinoma, carcinosarcoma and undifferentiated carcinoma, a staging infracolic omentectomy was added to the surgical procedure [23].

The definitive histopathological classification of a tumor, containing data about histotype, grade, lymphovascular space involvement (LVSI) and surgical stage was made by one of two pathologists experienced in gynecological malignancies according to the FIGO, 2014, and the World Health Organization, 2014 [24,25]. Based on final histopathological findings, patients were once again stratified into low- or high-risk groups and thereafter, decisions regarding adjuvant treatment, considering all the relevant factors, were made by the multidisciplinary board according to local guidelines.

After the completion of primary therapy, patients were transferred to a follow-up (FU) program consisting of an outpatient visit three or four times per year for the first 2 years, from years 3 to 5 on a six-month basis, and then once per year. The gynecological examination and pelvic US were an obligatory part of each visit. Blood sampling for marker assessment was performed once or twice during the follow-up and always when EC recurrence was suspected. The diagnosis of EC recurrence was confirmed either histologically or radiologically.

### 2.3. Clinical Data

The following data were prospectively recorded in the clinical database: age at the time of diagnosis, menopausal status, weight, height, body mass index (BMI), body surface area (BSA), renal functions (serum creatinine and glomerular filtration rate, calculated according to a chronic kidney disease epidemiology collaboration (CKD-EPI) equation), treatment data and date of the last follow-up visit or death (EC related/non-related).

### 2.4. Serum CA125, HE4, DJ1 and L1CAM Level Measurement

The sampling of the peripheral blood was performed under the standard procedure from the cubital vein, using 7.5 mL tubes of S-Monovette^®^ Serum Gel (Sarstedt) preoperatively, once or twice during the follow-up period and always when recurrence was suspected. The samples were transported to the Department of Clinical Biochemistry UH Brno, where the serum was separated by centrifugation, and the samples were analyzed either immediately (CA125, HE4) or stored frozen at −80 °C until analysis (DJ1 and L1CAM).

The quantitative assessments of L1CAM and DJ1 levels were performed by enzyme-linked immunosorbent assay (ELISA) using ELISA reader iMARK (Bio-Rad). For L1CAM, kit CN MBS 2023094 (MYBioSource, USA) was used. DJ1 serum levels were measured using kit CN CY-9050V2 (CircuLex MBL, UK). The serum concentrations of HE4 and CA125 were determined using quantitative, chemiluminescent microparticle immunoassay (CMIA) on the analyzer Architect i2000 (Abbott, Abbott Laboratories, USA). For CA125 measurement, the diagnostic set ARCHITECT Ca125 II (CN 2K45-24, Abbott) was used. HE4 serum level assessments were performed using the diagnostic set ARCHITECT HE4 (CN 2P51-25, Abbott).

### 2.5. Statistical Analysis

Categorical variables were summarized as absolute and relative frequencies and continuous variables as median, interquartile range (IQR), or range. Linear mixed-effects models were applied to evaluate the profiles of marker levels over time, and the impact of disease and patient characteristics on the levels. Models included the patient’s identification number as a random effect because one patient had more than one measurement. Original values were log-transformed due to their log-normal distribution for the purpose of the model. All tests were performed as two-sided at the significance level of alpha = 0.05. Analyses were done in R software.

## 3. Results

### 3.1. Patient Characteristics

A total of 65 patients with a median age of 65 years (30–65 years) and median BMI of 31.2 (17.3–45.7) were enrolled in the study. The majority of patients were diagnosed with an early stage disease (FIGO I–II, *n* = 58; 89%), while lymph nodes or distant metastases were diagnosed in 7 (11%) patients. In terms of histology, patients with low-grade endometrioid carcinoma predominated (*n* = 51; 79%). All the patients underwent a staging surgical procedure (*n* = 65; 100%), adjuvant radiotherapy was performed in 23 patients (35%) and chemotherapy in 7 (11%) patients. The median time between the preoperative blood sample and the first FU visit’s collection was 6.3 months (2.6–20.7 months), whereas between the first and second FU samples, 4.55 months (1.7–14.7 months). Detailed patients’ characteristics are shown in Table 1.

After a median follow-up time of 29.5 months, a total of five patients (8%) developed recurrent disease, with the time to progression between 7 and 16 months. One patient with initial FIGO stage II developed local recurrence, three had distant metastases (one of them initially staged FIGO IA, and two of them FIGO IVB) and one patient with initial FIGO stage IA developed both local recurrence and metastatic disease (Table 2). All four patients who developed distant metastases died from their disease (Table 1).

### 3.2. DJ1, L1CAM, CA125 and HE4 Serial Serum Levels in Correlation with Disease Status

**Figure 1:**
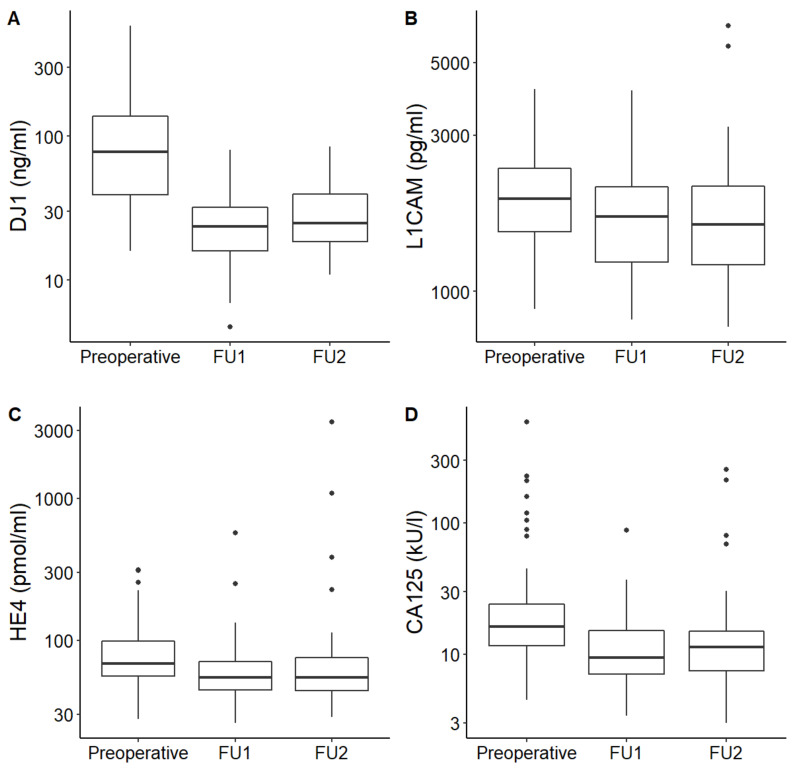
DJ-1 (**A**), L1CAM (**B**), HE4 (**C**) and CA125 (**D**) levels at three different time points: before surgery (preoperative), first follow-up collection (FU1) and second follow-up collection (FU2).

In all enrolled patients, median DJ-1, L1CAM, CA125 and HE4 serum levels fell after initial treatment and remained low for both subsequent follow-ups. Median serum levels of each marker were significantly higher at the time of diagnosis than afterwards in FU (*p* < 0.001 for DJ-1, L1CAM, CA125 and HE4, respectively). The serum levels of DJ-1, L1CAM, CA125 and HE4 at the time of diagnosis and during the follow-ups are shown in Table 3. Serial measurements are graphically illustrated in Figure 1.

**Figure 2:**
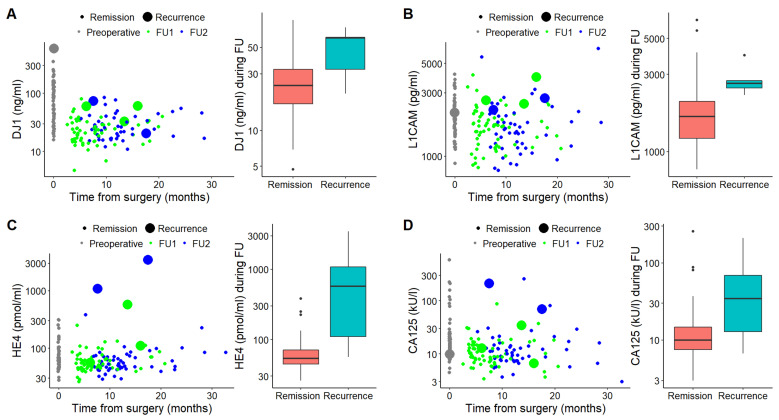
Charts displaying comparison of DJ-1 (**A**), L1CAM (**B**), HE4 (**C**) and CA125 (**D**) levels at the times of remission and recurrence. Scatter charts (left) display marker levels at the time of diagnosis (gray) and from the collections during follow-ups (first collection, green; second collection, blue). Large spots mean samples at the time of recurrence. Box plots (right) demonstrate marker levels at the times of remission and recurrence, respectively.

In patients with recurrent disease, serum DJ1, CA125 and HE4 levels were significantly higher at the time of recurrence compared to levels in disease-free patients (*p* = 0.035 for DJ1, *p* < 0.001 for CA125 and HE4, respectively) (Table 4, Figure 2A,C,D). Serum L1CAM levels were also higher in patients with recurrence; however, the patients’ profiles with or without recurrence did not differ significantly (*p* = 0.353) (Table 4, Figure 2B).

### 3.3. DJ1, L1CAM, CA125 and HE4 Serum Levels in Correlation with Patient-Related Characteristics

The correlation of DJ-1, L1CAM, CA125 and HE4 serum levels with age, weight, renal function and menopausal status are shown in Table 5. Median DJ1 serum levels were not affected by any of the observed patient-related characteristics. In contrast, median L1CAM levels were significantly higher in patients with age ≥60 years (*p* = 0.004), overweight (BMI ≥ 27 kg/m^2^, *p* = 0.002) and post-menopause (*p* = 0.010), respectively. None of the monitored patient-related parameters were related to either median CA125 or HE4 serum levels.

### 3.4. DJ1, L1CAM, CA125 and HE4 Serum Levels in Correlation with Tumor Clinicopathological Characteristics

In our study, DJ1 and L1CAM serum levels did not correlate with stage, histological type or risk of recurrence. Serum HE4 levels were statistically significantly higher in tumors with myometrial invasion ≥50% (*p* = 0.002), with lymph node involvement (*p* = 0.033), distant metastases (*p* = 0.021) and high risk of recurrence based on definitive histopathological findings (*p* = 0.02). There were also statistically significant different CA125 serum levels depending on the degree of myometrial invasion (lower levels in patients with invasion <50%, *p* = 0.009) and lymph node involvement (higher in case of lymph node metastases, *p* = 0.010). The detailed correlation of marker levels with clinicopathological features are shown in Table 6.

## 4. Discussion

Circulating tumor marker serum level determinations provide interpretable results that can significantly help to monitor treatment efficacy or early detection of recurrence in a broad array of solid tumors [26]. In EC patients, clear evidence of clinical benefit from circulating marker assessment has still been lacking and therefore, none of the circulating markers have become an integral part of EC patient management in clinical practice [23,27,28]. Studies that have been published so far have failed to demonstrate a higher proportion of recurrences diagnosed in asymptomatic patients, even when extensive FU was used consisting of not only gynecological examination, but also of serial CA125 measurements and examinations by imaging methods. The proportion of symptomatic recurrence remains 41–83% [29]. In general, there is still no consensus about FU regimens of EC patients after successful primary treatment [23,27,28]. Undoubtedly, the identification of a circulating marker detecting EC recurrence before the onset of symptoms, when lower tumor burden enables using a wider spectrum of therapeutic options, would have a significant impact on EC patient management.

In this pilot study, we aimed to evaluate the significance of the new circulating markers DJ1 and L1CAM in correlation with markers CA125 and HE4 in EC patients. We demonstrated that DJ1 and L1CAM serum levels were significantly higher at the time of EC diagnosis than levels collected after the initial treatment in disease-free patients (*p* < 0.001 for both markers, Figure 1A,B). Therefore, the dynamics of serial DJ1 and L1CAM serum levels correlate with disease status. The essential condition thus has been met for the use of DJ1 and L1CAM in monitoring of anticancer treatment efficacy. In concordance with previously published studies, we showed that CA125 and HE4 serum levels generally decreased after initial treatment as well (*p* < 0.001 for differences between levels collected preoperatively and during follow-up; Figure 1C,D) [12,13,30,31].

Despite the generally favorable prognosis of EC, 13–17% patients will develop recurrence, in most cases within three years after initial treatment [32]. While three-year survival following vaginal recurrence is ~73%, in case of pelvic or distant recurrence, it drops to less than 15% [33]. Moreover, patients with symptomatic recurrence survive for a significantly shorter time compared to patients whose recurrence is diagnosed in asymptomatic status [34]. In our study, a total of 5 patients (8%) developed recurrence after the median follow-up of 29.5 months. Three patients developed distant metastases despite initial stage FIGO I or II (Table 3). DJ1, CA125 and HE4 levels increased significantly at the time of recurrence (*p* = 0.035 for DJ1 and *p* < 0.001 for CA125 and HE4, respectively) (see Table 4 and Figure 2A,C,D). L1CAM serum levels at the time of recurrence increased as well, but statistical significance was not achieved (*p* = 0.353, Figure 2B and Table 4). The probable explanation for this fact could be a small number of patients with recurrent disease in our cohort. Furthermore, Tangen et al. demonstrated a correlation of L1CAM serum levels, with L1CAM tumor overexpression assessed immunohistochemically [20]. Similarly, Fogel et al. showed elevated L1CAM serum levels in patients with LCAM-positive EC in contrast to significantly lower levels in both healthy controls and patients with L1CAM-negative tumors [17]. In our study, there were only 10 patients with L1CAM-immunohistochemically positive tumors, and just one of them developed recurrent disease (unpublished data). Our results on CA125 and HE4 serum levels correlate with previously published studies providing evidence for the significance of serial monitoring of CA125 and HE4 serum levels for the detection of EC recurrence [12,13,35].

Undoubtedly, the identification of patient-related factors associated with health status that could affect marker serum levels is crucial for the use of circulating markers in clinical practice. In our study, DJ1 serum levels were affected only by the status of the disease (i.e., whether the samples were taken at the time of EC diagnosis, after treatment or at the time of recurrence). On the contrary, L1CAM serum levels also depended on age, weight and menopausal status (Table 5). Any association of CA125 or HE4 serum levels with any of the monitored parameters (Table 5) was not observed. The possible explanation of an inconsistency between our results and the previously described dependence of HE4 serum levels on renal function [36,37] might be a composition of the study cohort. Only one patient in a cohort assessable for HE4 had renal insufficiency, whereas the number of patients evaluable for L1CAM, DJ1 and CA125 having renal insufficiency was higher (see Table 5).

With respect to the comparison of DJ1 and L1CAM serum levels in relation to histological type, lymph node involvement, presence of distant metastases or risk of recurrence at the time of diagnosis, we did not demonstrate any statistically significant correlation (Table 6). Regarding DJ1, our data were consistent with the results of a study by Benati et al. Although this study observed higher DJ1 levels in EC patients (*n* = 45) compared to healthy controls (*n* = 29, *p* < 0.0001), the differences in serum levels between patients with an early stage (FIGO I, II; *n* = 38) or advanced disease (FIGO III, IV; *n* = 7) failed to reach statistical significance (*p* = 0.86) [16]. We did not confirm results of the study published by Di Cello et al. that demonstrated significantly higher DJ1 levels (*p* ≤ 0.05) in patients with a high-risk histological type (endometrioid carcinoma grade 3 or non-endometrioid carcinoma) than in patients with a low-risk histotype (endometrioid carcinoma grade 1 or 2) [15]. This discrepancy could be explained by the fact that the proportion of patients in our study with high-risk histotype was too small to reach statistical significance (*n* = 14; 22%) (see Table 6).

With respect to the correlation of L1CAM serum levels with tumor clinicopathological features, two studies have been published with conflicting results. Our results are consistent with the previous study published by Wojciechowski et al. (*n* = 35). Although the authors observed different L1CAM serum levels in EC patients compared to individuals with benign gynecological conditions, they were unable to demonstrate correlation among L1CAM serum levels and stage, histological type or tumor grading [19]. On the contrary, we did not confirm the correlation of L1CAM serum levels with the lymph node involvement demonstrated by Tangen et al. (*n* = 372; *p* = 0.048) [20]. L1CAM serum levels apparently depend on many patient-related variables, as we show in Table 5. In addition, the relation between L1CAM serum levels and L1CAM percentage tumor positivity (if any) has not been clearly defined yet [17]. Correlations of CA125 and HE4 serum levels with known prognostic factors in EC patients shown in our study (Table 6) are consistent with previously published data [10,12,13,38].

Preoperative knowledge of marker serum levels is only one part of the clinical complexity determining the risk of recurrence in EC patients [39]. Recently, the integrated genomic analyses performed by The Cancer Genome Atlas Research network (TCGA) proposed dividing EC into four groups with different clinical behaviors and prognoses [40,41,42]. This novel, molecular-based classification dramatically changed risk stratification and clinical management of EC patients [43,44,45]. Moreover, there are other promising methods that might be able to predict the behavior and pathological characteristics of EC such as metabolomics [46,47]. In this context, analyses of larger cohorts of patients taking into consideration other factors affecting EC prognosis such as the presence of POLE and p53 mutation, mismatch repair (MMR) status, the levels of L1CAM and estrogen/progesterone receptors’ positivity in the tumor, etc., need to be done for more specific assessment of the significance of circulating marker serum levels.

The strengths of this study include the fact that this is a cohort of fully staged EC patients from a real clinical practice with prospective data collection. To our best knowledge, this study is the first to analyze the dynamic changes of serial DJ1 and L1CAM serum levels in EC patients. In the case of DJ1, these are the first data supporting its potential role as a serum marker for the detection of recurrence in EC patients during the follow-up period after successful primary treatment. To date, none of the published studies on DJ1 and L1CAM serum levels have addressed this issue. A limitation of our study was that this was a relatively small cohort of EC patients with a shorter follow-up period and therefore a low recurrence rate, as it was a pilot study aimed at finding a reasonable preliminary evidence for future research on DJ1 and L1CAM as serum circulation tumor biomarkers in EC patients.

## 5. Conclusions

We demonstrated that DJ1 and L1CAM serum levels correlated with disease status in EC patients; they may therefore be potentially useful in clinical practice for monitoring the effects of treatment. Unlike the L1CAM marker, DJ1 serum levels were not affected by other factors associated with the health status of patients such as age or BMI. Further studies with longer follow-ups will be needed to definitively assess the benefit from monitoring of DJ1, L1CAM, CA125 and HE4 serum levels for the diagnosis of asymptomatic EC recurrence. At the time of EC diagnosis and in contrast to CA125 and HE4, DJ1 and L1CAM serum levels did not correlate with disease stage, histological type or risk of disease recurrence, leaving us unable to assess their prognostic significance in patients with endometrial cancer based on the limited pilot study cohort.

## Figures and Tables

**Table 1:** Characteristics of endometrial cancer patients.

Age (Years) Median (Min; Max)		65 (30; 85)
Menopausal status (*n*, %)	Pre-/perimenopausal	11 (17%)
	Postmenopausal	54 (83%)
Biometric data Median (min; max)	Weight (kg)	82 (50; 121)
	Height (cm)	165 (146; 176)
	BMI (kg/m^2^)	31.3 (17.3; 45.7)
	BSA (m^2^)	1.89 (1.5; 2.33)
Renal function, *n* = 64 Median (min; max)	Creat/S (umol/L)	69 (52; 134)
	CKD-EPI (mL/s)	1.33 (0.52; 1.81)
FIGO stage [25] (*n*; %)	I	50 (77%)
	II	8 (12%)
	III	5 (8%)
	IV	2 (3%)
Myometrial invasion (*n*; %)	<50%	48 (74%)
	≥50%	17 (26%)
Histology (*n*; %)	E G1-2	51 (79%)
	E G3, non-E	14 (21%)
Treatment (*n*; %)	HY and AE	65 (100%)
	PLN +/− PALN	20 (31%)
	RT	23 (35%)
	CHT	7 (11%)
Recurrence (*n*; %)	Local	2 (3%)
	Distant	3 (5%)
	No	60 (92%)
Time (months) Median (min; max) Surgery—FU1 FU1–FU2, *n* = 56 Dg–last FU		
		6.3 (2.6; 20.7)
		4.6 (1.7; 14.7)
		29.5 (13.7; 46.5)
Status at the end of FU (*n*; %)	Alive	58 (89%)
	Died of EC	4 (6%)
	Died (another cause)	3 (5%)

Abbreviations: *n* = number of patients, BMI = Body Mass Index, BSA = Body Surface Area, creat/S = creatinine serum level, CKD-EPI = glomerular filtration rate calculated according to chronic kidney disease epidemiology collaboration (CKD-EPI) equation, E = endometrioid, G = grading, HY = hysterectomy, AE = adnexectomy, PLN = pelvic lymphadenectomy, PALN = paraaortic lymphadenectomy, RT = radiotherapy, CHT = chemotherapy, FU = follow-up, FU1 = first follow-up blood sample, FU2 = second follow-up blood sample.

**Table 2:** Characteristics of patients with recurrent endometrial cancer.

Pts	Age	Histology	G	HY+AE	PLN/ PALN	RT	CHT	FIGO	Relapse	TTP (Months)
I	75	E	2	yes	no	yes	yes	IVB	Distant	8
II	71	E	3	yes	yes	yes	no	II	Local	16
III	72	E	2	yes	no	no	no	IA	Local, Distant	11
IV	55	E	2	yes	no	yes	yes	IVB	Distant	13
V	66	Non-E	3	yes	yes	no	yes	IA	Distant	7

Abbreviations: Pts = patients, E = endometrioid, non-E = non-endometrioid, HY = hysterectomy, AE = adnexectomy, PLN = pelvic lymphadenectomy, PALN = paraaortic lymphadenectomy, RT = radiotherapy, CHT = chemotherapy, TTP = time to progression.

**Table 3:** The measured DJ1, L1CAM, HE4 and CA125 levels in patients with EC.

		Time of Collection			*p* Value
		Preoperative	FU1	FU2	
DJ1 (ng/mL)	Valid *n*	64	65	50	<0.001
	Median (IQR)	78 (38.4–139)	23.5 (15.9–31.9)	25 (18.4–40.1)	
L1CAM (pg/mL)	Valid *n*	64	65	50	<0.001
	Median (IQR)	1919 (1519–2387)	1690 (1229–2087)	1602.5 (1195–2105)	
HE4 (pmol/L)	Valid *n*	49	65	56	<0.001
	Median (IQR)	68.2 (55.8–98)	54.5 (44.8–70.5)	54.7 (44.2–76.7)	
CA125 (kU/L)	Valid *n*	60	65	56	<0.001
	Median (IQR)	16.3 (11.4–24.2)	9.4 (7.1–15.2)	11.3 (7.5–15.4)	

Abbreviations: *n* = number of patients, IQR = interquartile range, FU1 = first follow-up blood sample, FU2 = second follow-up blood sample.

**Table 4:** DJ1, L1CAM, HE4 and CA125 follow-up serum levels in patients with or without recurrence of endometrial cancer.

		Serum Levels at Follow-Up		*p* Value
		Remission	Recurrence	
DJ1 (ng/mL)	Valid *n*	110	5	0.035
	Median (IQR)	23.9 (16.8–32.8)	60.1 (32.7–61)	
L1CAM (pg/mL)	Valid *n*	110	5	0.353
	Median (IQR)	1650.5 (1203–2050)	2630 (2474–2740)	
HE4 (pmol/L)	Valid *n*	116	5	<0.001
	Median (IQR)	53.5 (44.5–71.2)	572 (110–1083)	
CA125 (kU/L)	Valid *n*	116	5	<0.001
	Median (IQR)	10 (7.5–14.8)	34.4 (12.8–69.2)	

Abbreviations: *n* = number of measurements, IQR = interquartile range.

**Table 5:** DJ1, L1CAM, HE4 and CA125 preoperative serum levels according to age, weight, renal function and menopausal status.

			Valid *n*	Median (IQR)	*p* Value
DJ1 (ng/mL)	Age	<60 years	20	53.4 (31.4–103)	0.152
		≥60 years	44	88 (43–151.8)	
	Weight	BMI < 27 kg/m^2^	18	52.9 (25.5–121.9)	0.181
		BMI ≥ 27 kg/m^2^	48	86.2 (44.4–141.7)	
	Renal function	CKD-EPI ≥ 1 mL/s	56	83.4 (40.6–139)	0.604
		CKD-EPI < 1 mL/s	7	74.1 (24.7–352)	
	Menopausal status	Pre/perimenopausal	11	45.4 (24.4–142.4)	0.393
		Postmenopausal	53	84.9 (41.4–135.5)	
L1CAM (pg/mL)	Age	<60 years	20	1546.5 (1273.5–1872.5)	0.004
		≥60 years	44	2070 (1703.5–2447)	
	Weight	BMI < 27 kg/m^2^	16	1519.3 (1331.3–1804)	0.002
		BMI ≥ 27 kg/m^2^	48	2028 (1676.8–2507)	
	Renal function	CKD-EPI ≥ 1 mL/s	56	1889.5 (1503.5–2350.3)	0.105
		CKD-EPI < 1 mL/s	7	2247 (1878–3343.5)	
	Menopausal status	Pre/perimenopausal	11	1528 (1173–1928)	0.010
		Postmenopausal	53	2000 (1565–2424.5)	
HE4 (pmol/L)	Age	<60 years	15	65.7 (57.7–105)	0.765
		≥60 years	34	68.4 (53.1–98)	
	Weight	BMI < 27 kg/m^2^	13	65.7 (59.1–104.3)	0.570
		BMI ≥ 27 kg/m^2^	36	68.7 (53.6–94)	
	Renal function	CKD-EPI ≥ 1 mL/s	47	67.1 (53.1–98)	0.196
		CKD-EPI < 1 mL/s	1	161 (161–161)	
	Menopausal status	Pre/perimenopausal	8	63.4 (58.9–90.6)	0.468
		Postmenopausal	41	68.6 (53.1–98)	
CA125 (kU/L)	Age	<60 years	17	17.6 (9.5–24.9)	0.873
		≥60 years	43	15.8 (11.9–23.7)	
	Weight	BMI < 27 kg/m^2^	16	17.8 (13–24.8)	0.481
		BMI ≥ 27 kg/m^2^	44	15.4 (11.2–23.8)	
	Renal function	CKD-EPI ≥ 1 mL/s	54	17.3 (12.4–24.6)	0.128
		CKD-EPI < 1 mL/s	5	12.5 (9.9–15.8)	
	Menopausal status	Pre/perimenopausal	9	17.6 (15.1–24.9)	0.873
		Postmenopausal	51	15.8 (10.8–23.8)	

Abbreviations: *n* = number of patients, IQR = interquartile range, BMI = Body Mass Index, CKD-EPI = glomerular filtration rate calculated according to the chronic kidney disease epidemiology collaboration (CKD-EPI) equation.

**Table 6:** DJ1, L1CAM, HE4 and CA125 serum levels in correlation with both clinical and pathological features of endometrial cancer.

			**Valid *n***	**Median (IQR)**	***p*** **Value**
DJ1 (ng/mL)	Histological type	E G1-2	50	83.4 (41.4–151.5)	0.191
		E G3, non-E	14	69 (22.1–94.5)	
	Myometrial invasion	<50%	47	63.9 (35.4–135.5)	0.331
		≥50%	17	91.1 (51.9–151.5)	
	LN involvement	No	60	78 (38.4–145.2)	0.688
		Yes	4	73.2 (36.4–111.3)	
	Distant metastasis	No	62	83.4 (37.1–142.4)	0.714
		Yes	2	56.7 (53.1–60.3)	
	Definitive risk	Low ^1^	34	57.8 (39.7–147.9)	0.941
		High ^2^	30	86.2 (37.1–128)	
L1CAM (pg/mL)	Histological type	E G1-2	50	1889.5 (1523.5–2418)	0.620
		E G3, non-E	14	2132.5 (1460–2345.5)	
	Myometrial invasion	<50%	47	1969 (1528–2355)	0.721
		≥50%	17	1680 (1515–2418)	
	LN involvement	No	60	1919 (1519.3–2389.8)	0.945
		Yes	4	2055.5 (1586.5–2381.8)	
	Distant metastasis	No	62	1919 (1523.5–2355)	0.772
		Yes	2	2200.5 (1515–2886)	
	Definitive risk	Low ^1^	34	1948.5 (1580–2355)	0.687
		High ^2^	30	1837.75 (1469–2418)	
HE4 (pmol/mL)	Histological type	E G1-2	38	65 (51.4–98)	0.276
		E G3, non-E	11	76.2 (60.2–153.7)	
	Myometrial invasion	<50%	39	63.7 (51.4–79.2)	0.002
		≥50%	10	148.4 (68.6–255)	
	LN involvement	No	45	65.7 (53.1–89.6)	0.033
		Yes	4	148.4 (105.8–189.4)	
	Distant metastasis	No	47	67.1 (53.1–90)	0.021
		Yes	2	284.5 (255–314)	
	Definitive risk	Low ^1^	28	61.9 (48.3–79.9)	0.020
		High ^2^	21	76.2 (61.1–153.7)	
CA125 (kU/L)	Histological type	E G1-2	47	15.8 (12.4–23.8)	0.837
		E G3, non-E	13	17.9 (10.8–24.7)	
	Myometrial invasion	<50%	45	14.9 (10.5–20)	0.009
		≥50%	15	23.8 (16.9–119)	
	LN involvement	No	56	15.8 (10.7–23.6)	0.010
		Yes	4	192.4 (88.2–407.4)	
	Distant metastasis	No	58	16 (10.8–23.8)	0.104
		Yes	2	116.7 (23.4–210)	
	Definitive risk	Low ^1^	32	15.3 (10.2–19.8)	0.135
		High ^2^	28	19 (12.2–41.4)	

Abbreviations: *n* = number of patients, IQR = interquartile range. ^1^ Low definitive risk = endometrial carcinoma G1-2 AND myometrial invasion <50% AND LVSI negative (i.e., adjuvant treatment not recommended) ^2^ High definitive risk = criteria for low risk not met (i.e., adjuvant treatment recommended).

## Data Availability

The data presented in this study are available from the corresponding author upon reasonable request.
